# Bone marrow-derived mesenchymal stem/stromal cells reverse the sensorial diabetic neuropathy via modulation of spinal neuroinflammatory cascades

**DOI:** 10.1186/s12974-018-1224-3

**Published:** 2018-06-22

**Authors:** Afrânio Ferreira Evangelista, Marcos André Vannier-Santos, Gessica Sabrina de Assis Silva, Daniela Nascimento Silva, Paulo José Lima Juiz, Carolina Kymie Vasques Nonaka, Ricardo Ribeiro dos Santos, Milena Botelho Pereira Soares, Cristiane Flora Villarreal

**Affiliations:** 1 Gonçalo Moniz Institute, Oswaldo Cruz Foundation-FIOCRUZ, Salvador, BA CEP 40296-710 Brazil; 2 Oswaldo Cruz Institute, Oswaldo Cruz Foundation-IOC-FIOCRUZ, Rio de Janeiro, RJ CEP 21040-900 Brazil; 3 Pharmacy College, Federal University of Bahia, Salvador, BA CEP 40170-290 Brazil; 4 Center of Biotechnology and Cell Therapy, São Rafael Hospital, Salvador, BA CEP 41253-190 Brazil; 5 Federal University of Recôncavo of Bahia, Feira de Santana, BA CEP 44042-280 Brazil

**Keywords:** Stem cells, Sensory neuropathy, Diabetes, Spinal cord, Neuroinflammation, Galectin-3

## Abstract

**Background:**

Diabetic neuropathy (DN) is a frequent and debilitating manifestation of diabetes mellitus, to which there are no effective therapeutic approaches. Mesenchymal stem/stromal cells (MSC) have a great potential for the treatment of this syndrome, possibly through regenerative actions on peripheral nerves. Here, we evaluated the therapeutic effects of MSC on spinal neuroinflammation, as well as on ultrastructural aspects of the peripheral nerve in DN-associated sensorial dysfunction.

**Methods:**

C57Bl/6 mice were treated with bone marrow-derived MSC (1 × 10^6^), conditioned medium from MSC cultures (CM-MSC) or vehicle by endovenous route following the onset of streptozotocin (STZ)-induced diabetes. Paw mechanical and thermal nociceptive thresholds were evaluated by using von Frey filaments and Hargreaves test, respectively. Morphological and morphometric analysis of the sciatic nerve was performed by light microscopy and transmission electron microscopy. Mediators and markers of neuroinflammation in the spinal cord were measured by radioimmunoassay, real-time PCR, and immunofluorescence analyses.

**Results:**

Diabetic mice presented behavioral signs of sensory neuropathy, mechanical allodynia, and heat hypoalgesia, which were completely reversed by a single administration of MSC or CM-MSC. The ultrastructural analysis of the sciatic nerve showed that diabetic mice exhibited morphological and morphometric alterations, considered hallmarks of DN, such as degenerative changes in axons and myelin sheath, and reduced area and density of unmyelinated fibers. In MSC-treated mice, these structural alterations were markedly less commonly observed and/or less pronounced. Moreover, MSC transplantation inhibited multiple parameters of spinal neuroinflammation found in diabetic mice, causing the reduction of activated astrocytes and microglia, oxidative stress signals, galectin-3, IL-1β, and TNF-α production. Conversely, MSC increased the levels of anti-inflammatory cytokines, IL-10, and TGF-β.

**Conclusions:**

The present study described the modulatory effects of MSC on spinal cord neuroinflammation in diabetic mice, suggesting new mechanisms by which MSC can improve DN.

## Background

Diabetes mellitus is a highly debilitating disease that affects humans, with an estimated global prevalence of 6% [1]. Among the several complications that contribute to the reduced life quality and life expectancy of diabetic patients, diabetic neuropathy (DN) is the most frequent and recognized nervous system pathology, affecting approximately 50% of patients with both type 1 and type 2 diabetes [2]. Axonal degeneration, demyelination, and disordered repair are observed in the nerves of patients with diabetes, affecting both myelinated and unmyelinated, as well as large and small fibers [3]. Clinical manifestations of DN include painful neuropathic symptoms, such as spontaneous pain, hyperalgesia and allodynia, and sensory loss, resulting in foot ulcerations and amputations [4].

Glycemic control can slow, but not completely prevent, the progression of DN [3], and thus, therapies aiming to relieve sensory symptoms are essential. Additionally, the available analgesic drugs appear to be relatively ineffective in controlling neuropathic pain associated with DN [5]. Currently, there are no drugs available that can restore nerve function, and the usual therapeutic strategies for diabetic neuropathic pain are limited to palliative analgesic effects [6].

Successful control of DN is linked to the establishment of new disease-modifying therapeutic approaches. In this context, cell-based therapies represent a promising alternative. Cellular therapies have shown favorable results and can be considered as a potential approach in treating neuropathic pain [7]. The cell transplantation strategy for neuropathic pain treatment is focused on cell-based analgesia and neuroprotective/regenerative potential. In this setting, stem cells can represent not only a treatment for pain but also a method aimed at repairing the damaged nervous system [8]. Recent studies have shown that transplantation of progenitor/stem cells, such as endothelial progenitor cells and mesenchymal stem/stromal cells (MSC), ameliorates diabetic neuropathy in experimental diabetes [9–14]. Shibata et al. reported that intramuscularly transplanted MSC improves sciatic nerve conduction velocity, sciatic nerve blood flow, as well as increases density of small vessels in the muscle of streptozotocin (STZ)-induced diabetic rats [9]. These authors suggested that the beneficial effects are mediated by paracrine actions of locally released angiogenic factors, such as vascular endothelial growth factor and basic fibroblast growth factor. Due to the key role of ischemia and decreased nerve blood flow in the pathophysiology of diabetic neuropathy [15], the lack of angiogenic factors has been regarded as an important mechanism of DN [16]. Considering that MSC secrete neurotrophic and angiogenic factors [17, 18], these were the first candidates to explain the efficacy of cell therapy for DN, as suggested by a number of studies [9–11, 13]. In addition, the contribution of immunosuppressive and anti-inflammatory effects of MSC on peripheral nerves in STZ-induced DN has also been proposed [12, 19].

Although there is strong evidence of the importance of peripheral nerve pathological processes in DN pathogenesis, there is now emerging evidence of the involvement of the central nervous system in diabetic neuropathy. Multiple biochemical and anatomical alterations in the central nervous systems have been associated with the development and maintenance of DN [20]. In contrast to traumatic neuropathy, during DN, the spinal sensory neurons are not principally driven by input from primary afferent neurons because sensory inputs to the spinal cord decrease rather than increase in diabetes [21, 22]. Hyperglycemia and the resulting oxidative stress affect the local microenvironment in the spinal cord, promoting activation of glial cells [22]. In turn, activated spinal glial cells induce a series of alterations, such as activation of intracellular signaling pathways and neuroinflammation, which directly influence the establishment of sensorial neuropathy [22–24]. Considering this scenario, the present study was designed to investigate the hypothesis that regulation of spinal neuromodulator pathways, underlying the maintenance of diabetic neuropathy, contributes to MSC-induced beneficial effects on sensorial dysfunction during DN. In addition, the effects of MSC on ultrastructural aspects of the peripheral nerve were also investigated.

## Methods

### Bone marrow-derived mesenchymal cell (MSC) culture and conditioned medium preparation

Mesenchymal stem cells were obtained from the bone marrow of femurs and tibiae of GFP (green fluorescent protein) transgenic C57Bl/6 mice. Bone marrow samples were diluted in Dulbecco’s modified Eagle’s medium (DMEM; Gibco, Carlsbad, CA, USA), and the mononuclear cell fraction was obtained by Ficoll-Hypaque gradient (Sigma, St Louis, MO, USA), after centrifugation at 400×*g* for 30 min at 20 °C. The interface containing mononuclear cells was collected in individualized tubes and washed twice in incomplete DMEM. Mononuclear cells were resuspended in DMEM medium supplemented with 2 mM L glutamine, 1 mM sodium piruvate, 50 μg/mL gentamycin, and 10% fetal bovine serum (all reagents were acquired from Sigma) and cultured at the density of 10^5^ cells/cm^2^ in polystyrene plates. Cell cultures were maintained at 37 °C with 5% CO_2_. The cells were expanded during approximately five passages, and when 90% confluence was reached, the cells were detached using 0.25% trypsin (Invitrogen/Molecular Probes, Eugene, OR, USA) and expanded in new culture bottles (9 × 10^3^ cells/cm^2^). The identity of MSC was confirmed on the basis of morphological criteria, plastic adherence, and specific surface antigen expression: CD90 (+), CD44 (+), Sca-1 (+), CD45(−), CD34 (−), and CD11b (−). Differentiation ability of MSC was also evaluated after induction using specific media, as previously described [25]. Oil Red, Alizarin Red, and Alcian Blue stainings (Sigma) were used to assess adipogenic, osteogenic, and chondrogenic differentiation, respectively.

Conditioned medium (CM) was obtained from MSC cultures (CM-MSC), as previously described [26]. MSC (7 × 10^6^, five passages) were washed three times with phosphate-buffered saline (PBS) and transferred to a serum-free DMEM culture medium during 24 h. Then, CM was concentrated 15 times by centrifugation at 4000 g for 15 min at 13 °C, using ultrafiltration units (Amicon Ultra-PL 10, Millipore, Bedford, MA, USA). Filter units were used only one time to avoid membrane saturation. Concentrated CM-MSC were then sterilized on 0.22 μm filters (Millipore) and stored at − 80 °C until used. CM-MSC was divided into aliquots of 700 μL before freezing to avoid repeated freeze/thaw cycles. The mean protein concentration of CM-MSC was of 1.5–1.8 mg/ml, and there was no difference between fresh and frozen CM-MSC. Serum-free DMEM, centrifuged and filtered, was used as control medium (vehicle group).

### Animals

Experiments were performed on male C57Bl/6 mice (20–25 g) obtained from the Animal Facilities of Instituto Gonçalo Moniz/FIOCRUZ (Brazil). MSC were obtained from male GFP transgenic C57Bl/6 mice. Animals were housed in temperature-controlled rooms (22–25 °C), under a 12:12-h light-dark cycle, with access to water and food, ad libitum. All behavioral tests were performed between 8:00 a.m. and 5:00 p.m., and animals were only tested once. Animal care and handling procedures were in accordance with the National Institutes of Health guidelines for the care and use of laboratory animals (NIH, 8023) and the Institutional Animal Care and Use Committee FIOCRUZ (CPqGM 025/2011). Every effort was made to minimize the number of animals used and to avoid any unnecessary discomfort.

### Diabetic neuropathy model

Diabetes was induced by intraperitoneal (i.p.) injection of streptozotocin (80 mg/kg in citrate buffer, pH 4.5) for three consecutive days [14]. The control group received citrate buffer in the place of streptozotocin. Blood glucose levels were determined in blood samples from the tail vein using ACCU-CHEK glucose sticks. Mice were considered diabetic if glycemia values exceeded 250 mg/dL. Pain-like behaviors were assessed throughout the experimental period to confirm the development of the DN.

### Assessment of diabetic sensorial neuropathy by behavioral assays

Sensorial parameters of DN were assessed throughout the experimental period by using the established behavioral assays that evaluate mechanical and thermal nociceptive thresholds [27, 28]. Behavioral tests were performed in blind fashion. Withdrawal threshold to mechanical stimulation was measured with von Frey filaments (Stoelting; Chicago, IL, USA). In a quiet room, mice were placed in acrylic cages (12 × 10 × 17 cm) with wire grid floor, allowing full access to the ventral aspect of the hind paws, 40 min before the beginning of the test. A logarithmic series of nine filaments were applied to the plantar surface of the ipsilateral hind paw to determine the threshold stiffness required for 50% paw withdrawal according to the non-parametric method of Dixon, as described by Chaplan and collaborators [28]. A positive response was characterized by the removal of the paw followed by clear flinching movements. The development of DN was characterized by mechanical allodynia, indicated by the reduction of the paw withdrawal threshold (in grams).

Withdrawal threshold to heat stimulation was determined using the Plantar Test (Hargreaves Apparatus, Ugo Basile Biological Instruments, Gemonio, Italy) as previously described [27]. Similar to the von Frey test, mice underwent an acclimatization period before the beginning of the test. An infra-red light source was placed under the glass floor and positioned at the center of the hind paw of mice. On paw withdrawal, a photo-cell automatically shut off the heat source and recorded the time to withdrawal. To avoid thermal injury, there was an upper cutoff limit of 20 s after which the heating was automatically terminated. The stimulation was applied three times with intervals of at least 5 min. The averaged threshold from these three trials was recorded as the thermal nociception threshold. Heat hypoalgesia was indicated by the increase of the paw withdrawal threshold (in seconds).

### Motor function assay

To evaluate the motor performance, mice were submitted to the rota-rod test, as previously described [29]. The rota-rod apparatus (Insight, Ribeirão Preto, Brazil) consisted of a bar with a diameter of 3 cm, subdivided into five compartments. On the test day, mice from different experimental groups were placed on the rotating rod (eight revolutions per min) and the falling avoidance was measured for up to 120 s. Mice treated with diazepam (10 mg/kg; Cristália, Itapira, SP, Brazil), the test reference drug, were placed on a rotating rod 1 h after treatment. The results were analyzed as the average time (s) the animals remained on the rota-rod in each group.

### Experimental design

Mice were divided into the following groups (*n* = 6): control non-diabetic group, diabetic neuropathy plus control treatment (STZ + saline), diabetic neuropathy plus MSC treatment (STZ + MSC), diabetic neuropathy plus CM-MSC treatment (STZ + CM-MSC), and diabetic neuropathy plus CM-MSC treatment control (STZ + vehicle). Nociceptive tests (von Frey and Plantar Test) were performed at baseline and daily after diabetic neuropathy induction. Four weeks following induction, and after the establishment of behavioral neuropathic pain as assessed by nociceptive tests, mice were transplanted via tail vein injection with 1 × 10^6^ cells/mouse in a final volume of 100 μL. The number of transplanted MSC was defined based on previous work [14]. The STZ + saline and STZ + vehicle groups received an endovenous injection (100 μL) of saline or vehicle (serum-free DMEM, centrifuged and filtered), respectively. Motor performance and body weight were recorded weekly to assess general toxicity. Two and 8 weeks after treatments, mice were sacrificed for biological sampling. For the transplanted MSC tracking study, mice were sacrificed 24 h, 1 week or 3 weeks after MSC injection, for biological sampling.

### Morphological and morphometric analysis of sciatic nerve

Eight weeks after treatments (12 weeks after the neuropathy induction), mice were euthanized and sciatic nerve samples (± 1 cm) were collected, processed and subjected to morphological and morphometric analysis by light microscopy and transmission electron microscopy. The samples were fixed in 2.5% glutaraldehyde (grade I, Sigma) in 0.1 M sodium cacodylate buffer overnight; washed in cacodylate buffer; post-fixed in 1% osmium tetroxide (Sigma), 0.8% potassium ferricyanide, and 5 mM CaCl_2_ in the same buffer for 60 min; serially dehydrated using graded acetone; and embedded in Poly/Bed resin (Polysciences, Warrington, PA, USA).

Transverse sections (1 μm thick) were stained with 1% toluidine blue and examined by light microscopy. Images of semi-thin sections were captured and examined using the software Image-Pro Plus 7.01 (MediaCybernetics, Rockville, MD, USA). Morphometric parameters of myelinated fibers, such as axonal diameter, fiber diameter, myelin sheath thickness, percentage of abnormal fibers (fibers with irregular shapes, infoldings, or compacted myelin), and *G* ratio values were obtained, as described previously [30–32]. For ultrastructural analysis of unmyelinated fibers, ultrathin sections were stained with 5% uranyl acetate for 30 min and 3% lead citrate for 5 min and observed in JEOL electron microscope (JEM - 1230). Morphological and morphometric evaluation of unmyelinated fibers was performed, as previously described [33, 34].

### Cytokine measurement by ELISA

For the measurement of cytokine levels, the spinal cords were collected 2 and 8 weeks after treatments, in mice terminally anesthetized with halothane vaporized in 95% O_2_ and 5% CO_2_ from each experimental group. The L4–L5 spinal segments were removed and rapidly frozen and stored at − 80 °C. Samples were homogenized in ice-cold phosphate-buffered saline (PBS; 100 mg tissue/mL) to which 0.4 M NaCl, 0.05% Tween 20, and protease inhibitors (0.1 mM PMSF, 0.1 mM benzethonium chloride, 10 mM EDTA, and 20 KI aprotinin A/100 mL) were added (Sigma). The samples were centrifuged for 10 min at 3000 *g*, and supernatant aliquots were frozen at − 80 °C for later quantification. Tumor necrosis factor-α (TNF-α), interleukin-1β (IL-1β), interleukin-10 (IL-10), and transforming growth factor-β (TGF-β) levels were estimated using commercially available immunoassay ELISA kits for mice (R&D System, Minneapolis, MN, USA), according to the manufacturer’s instructions. The results are expressed as picograms of cytokine per milligram of protein.

### Real-time PCR

The transcription of catalase, superoxide dismutase, glutathione peroxidase, and Nrf2 genes was evaluated by real-time quantitative polymerase chain reaction (qRT-PCR) in mouse spinal cord at the conclusion of the experimental period (8 weeks after treatments). Total RNA was extracted from L4–L5 spinal segments with TRIzol reagent (Invitrogen, Carlsbad, CA, USA) and the concentration determined by photometric measurement. A High-Capacity cDNA Reverse Transcription Kit (Applied Biosystems, Foster City, CA, USA) was used to synthesize cDNA from 1 μg of RNA, according to the manufacturer’s recommendations. Synthesis of cDNA and RNA expression analysis was performed by real-time PCR using TaqMan Gene Expression Assay for *Cat* (Mm00437992_m1), *Sod1* (Mm01344233_g1), *Gpx1* (Mm00492427_m1), and *Nrf2* (Mm00477784_m1). A no-template control (NTC) and no-reverse transcription controls (No–RT) were also included. All reactions were run in duplicate on an ABI7500 Sequence Detection System (Applied Biosystems) under standard thermal cycling conditions. The mean Ct (cycle threshold) values from duplicate measurements were used to calculate expression of the target gene, with normalization to an internal control––*Gapdh* (Mm99999915_g1), using the 2–DCt formula. Experiments with coefficients of variation greater than 5% were excluded. For transplanted MSC tracking, the transcription of GFP gene was evaluated in the spinal cord, sciatic nerve, dorsal root ganglion, spleen, and lung of mice 24 h, 1 and 3 weeks after MSC treatment. The mean Ct (cycle threshold) values were used to calculate expression of GFP, normalized to *Gapdh*, using the cycle threshold method of comparative PCR [35].

### Estimation of nitrite and lipid peroxidation

At the end of the experimental period (8 weeks after treatments), the spinal cords were collected. L4–L5 spinal segments were rinsed with ice-cold saline and homogenized in chilled phosphate buffer (pH 7.4), then used to determine lipid peroxidation and nitrite estimation. The malondialdehyde (MDA) content, a marker of lipid peroxidation, was assayed in the form of thiobarbituric acid-reactive substances, as previously described [36]. Briefly, 0.5 ml of homogenate and 0.5 mL of Tris–HCl were incubated at 37 °C for 2 h. After incubation, 1 ml of 10% trichloroacetic acid was added and centrifuged at 1000 g for 10 min. To 1 mL of supernatant, 1 mL of 0.67% thiobarbituric acid was added and the tubes were kept in boiling water for 10 min. After cooling, 1 mL double distilled water was added and absorbance was measured at 532 nm. Thiobarbituric acid-reactive substances were quantified using an extinction coefficient of 1.56 × 10^5^ M^−1^ cm^−1^ and were expressed as nanomoles of malondialdehyde per milligram of protein. Nitrite was estimated in the spinal cord homogenate using the Griess reagent and served as an indicator of nitric oxide production [37]. Next, 500 μL of Griess reagent (1:1 solution of 1% sulphanilamide in 5% phosphoric acid and 0.1% napthaylamine diamine dihydrochloric acid in water) was added to 100 μL of homogenate, and absorbance was measured at 546 nm. Nitrite concentration (μg/ml) was calculated using a standard curve for sodium nitrite.

### Confocal immunofluorescence analyses in the spinal cord of mice

At the conclusion of the experimental period (8 weeks after treatments), mice were anesthetized with halothane vaporized in 95% O_2_ and 5% CO_2_ and transcardially perfused with saline solution, followed by 4% paraformaldehyde (PFA) solution (Electron Microscopy Sciences, Hatfield, PA, USA) in 0.01 M phosphate-buffered saline (PBS). The spinal cords were collected, post-fixed overnight at 4 °C in 4% PFA, cryoprotected for 48 h at 4 °C in 30% sucrose (Merck, Whitehouse Station, NJ, USA) in PBS, embedded in optimal cutting temperature embedding compound (O.C.T., Sakura Tissue-Tek), and frozen at − 80 °C. Transverse spinal cord sections (4 μm thick) were obtained, fixed in 4% PFA for 10 min, and washed in PBS twice for 5 min. Non-specific binding was blocked by incubating the sections in 5% BSA in PBS for 1 h, followed by incubation overnight with primary antibodies solution containing rat anti-glial fibrillary acidic protein––GFAP (1:200, Zymed, Thermo Fisher Scientific, Waltham, MA, USA), goat anti-Iba1, and rabbit anti-galectin-3, diluted in 1% BSA in PBS. Sections were then incubated with the secondary antibodies, anti-rat IgG Alexa Fluor 594 conjugated for GFAP (1:800; Molecular Probes, Carlsbad, CA, USA), anti-goat IgG Alexa Fluor 488 conjugated for Iba1 (1:600; Molecular Probes), or anti-rabbit IgG Alexa Fluor 568 conjugated for galectin-3 (1:100; Molecular Probes) during 1 h at room temperature. Nuclei were stained with Vectashield mounting medium with DAPI, 4′,6-diamidino-2-phenylindole (Vector Laboratories, Burlingame, CA, USA). Quantitative analysis, expressed as percentage of immuno-positive area, was performed using a confocal laser scanning A1R microscope (Nikon, Tokyo, Japan). The area displaying immunoreactive staining for Iba1, GFAP, or gal-3 in the superficial spinal dorsal horn (laminae I–III) was measured using Image-Pro Plus v. 7.01 (Media Cybernetics, Rockville, MD, USA). The area of the ipsilateral superficial spinal dorsal horn was also calculated. The ratio of the above areas was used as the percentage area density of Iba1, GFAP, or gal-3 [38].

### Statistical analyses

All data are presented as means ± standard error of the mean (S.E.M) of measurements made on six animals in each group. Behavioral data were analyzed using two-way ANOVA (group and time) followed by Bonferroni’s multiple comparisons. For morphometric analysis, Shapiro Wilk test was performed, and because all data were negative for normality, Kurskal Wallis followed by Dunns post-test was used. Remaining data were analyzed using one-way ANOVA followed by Tukey’s post-test. All data were analyzed using the GraphPad Prism v.5.0 software (GraphPad Inc.). Differences were considered statistically significant for *p* values < 0.05.

## Results

### MSC transplantation reduces the sensorial dysfunction in diabetic neuropathic mice

**Fig. 1:**
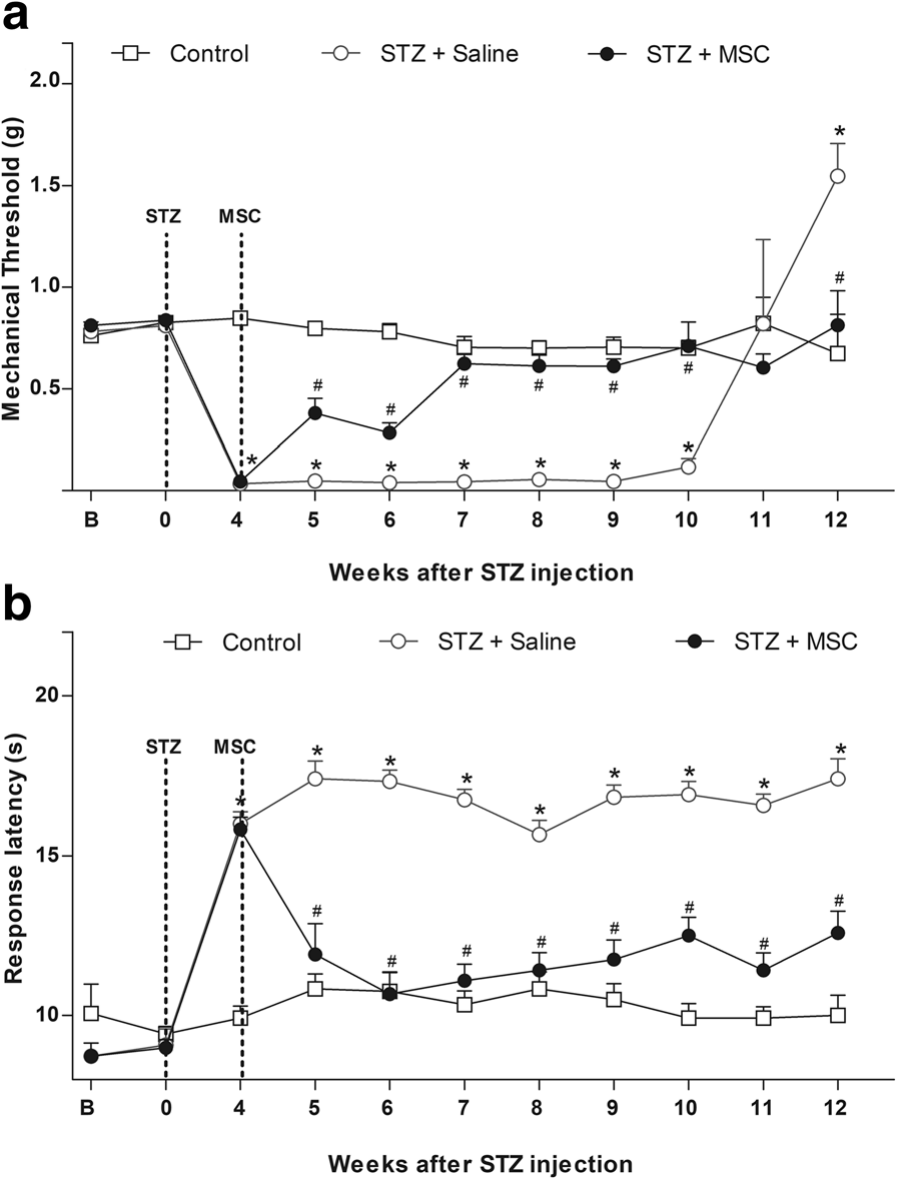
Effect of MSC on pain-like behaviors of mice with diabetic neuropathy. **a** Mechanical nociceptive thresholds: ordinates represent the filament weight (g) in which the animal responds in 50% of presentations. **b** Thermal nociceptive threshold: the axis of ordinates represents the time (seconds) the animal takes to withdraw its paw. The nociceptive thresholds were assessed in the paw of each mouse before (**b**) and after the model induction with streptozotocin (STZ; week 0). Control group represents mice without diabetic neuropathy, in which saline was administered instead of streptozotocin. Four weeks after induction, mice were treated via endovenous route with bone marrow-derived mesenchymal cells (STZ + MSC; 1 × 10^6^/100 μL) or vehicle (STZ + saline; 100 μL). Data are expressed as means ± SEM; *n* = 6 mice per group. *Statistical significance relative to the control group (*p* < 0.001); ^#^Statistical significance relative to the STZ + saline group (*p* < 0.001), as determined by two-way ANOVA followed by Bonferroni post-test

Behavioral testing was performed at baseline and daily, after the model induction, to evaluate the effects of MSC transplantation on measurable sensorial parameters of STZ-induced diabetic neuropathy. All mice survived until the end of the study. There were no signs of distress, motor disability, or general toxicity. STZ treatment induced sensory neuropathy associated with mechanical allodynia (Fig. 1a) and heat hypoalgesia in mice (Fig. 1b), without causing motor impairment, as assessed by the rota-rod test (data not shown).

Behavioral signs of sensory neuropathy were evident, 1 week after the diabetes model induction. Heat hypoalgesia persisted during the experimental period of 12 weeks (*p* < 0.001), while mechanical allodynia was maintained for 10 weeks, at which time diabetic mice showed a gradual loss of mechanical sensitivity (*p* < 0.001). To determine whether MSC induce therapeutic effects in diabetic sensory neuropathy, mice were treated with MSC (1 × 10^6^, 100 μL) or vehicle (100 μL) 4 weeks after diabetes induction, when sensorial neuropathy was fully established. One week after administration, neuropathic mice treated with MSC exhibited antinociceptive effect to mechanical stimuli (Fig. 1a, *p* < 0.05). The antinociceptive effect of MSC was progressive, peaking 3 weeks after treatment, when a complete reversion of the mechanical allodynia was achieved (*p* < 0.001). Importantly, the progression of sensory neuropathy, indicated by the late loss of mechanical sensitivity, was completely prevented in MSC-treated mice. Additionally, the MSC treatment also reverted the heat hypoalgesia of neuropathic mice from 7 days after administration until the end of the evaluation period (Fig. 1b, *p* < 0.001).

### MSC restore the morphological pattern of the sciatic nerve of mice with diabetic neuropathy

**Fig. 2:**
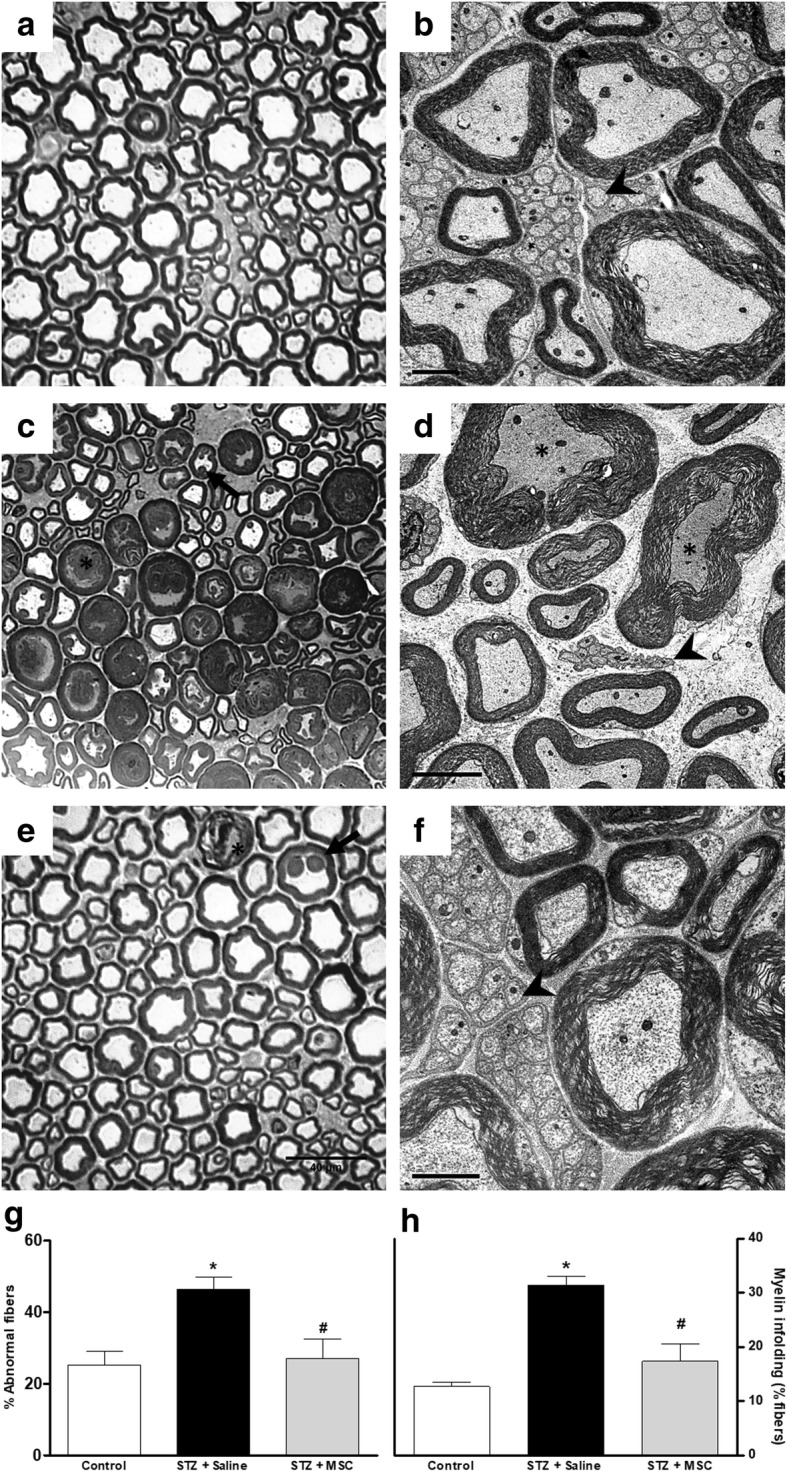
Effect of MSC on the morphology of sciatic nerve from mice with diabetic neuropathy. Representative photomicrographs of sciatic nerve cross-sections from non-diabetic mice (panel **a**, control group), diabetic mice treated with saline (panel **c**), and diabetic mice treated with MSC (1 × 10^6^, panel **e**), 12 weeks after the neuropathy induction. Light microscopy revealed that sciatic nerve from diabetic mice (**c**) had large myelin fibers with axonal atrophy, loose myelin sheath (*), and myelin with infoldings into to the axoplasm (arrow). Panel **e** shows that sciatic nerve from MSC-treated neuropathic mice presented myelin fibers of various calibers with normal morphology. Scale bar = 40 μm. Electron microscopy of sciatic nerve cross-sections from non-diabetic mice (panel **b**, control group), diabetic mice treated with saline (panel **d**), and diabetic mice treated with MSC (1 × 10^6^, panel **f**). Analysis of ultrastructural aspects of the sciatic nerve shows in **b** myelin fibers with varying sizes and proportional myelin sheath, including numerous unmyelinated fibers; in **d** few unmyelinated fibers (arrowhead) and the presence of atrophic axons with loose myelin sheath (*); and in **f** myelinated fibers with myelin sheath of varying diameters and a large amount of unmyelinated fibers (arrowhead). Scale bar = 2 μm. Panels **g** and **h** show the percentage of abnormal myelinic fibers and fibers with myelin infoldings, respectively. Data are expressed as means ± SEM; *n* = 3 mice per group. *Statistically significant as compared to the control group (*p* < 0.05). ^#^Statistically significant as compared to the STZ + saline group (*p* < 0.05). One-way ANOVA followed by Tukey’s multiple comparison test

Since diabetic neuropathy is associated with morphological alterations in the peripheral nerves, morphological analysis of myelinated and unmyelinated fibers of the sciatic nerve were performed at the conclusion of the experimental period by light and electron microscopy. Data from light microscopy showed that sciatic nerves from non-diabetic control mice present myelinated fibers of varying diameters, regular contours, intact myelin sheaths, and thickness proportional to the diameter of their axons (Fig. 2a). The ultrastructural evaluation of sciatic nerves from non-diabetic mice by electron microscopy showed axons of myelinated fibers surrounded by a typical myelin sheath and the presence of numerous unmyelinated fibers, evenly distributed in endoneural space (Fig. 2b). In diabetic mice, myelinated fibers with axonal atrophy and invasion of the myelin sheath were observed (infoldings; Fig. 2c). In addition, large diameter myelinated fibers in axonal atrophy process with loosening of the myelin sheath and apparent diminution of unmyelinated fiber numbers were found in sciatic nerves of diabetic mice (Fig. 2d). The qualitative assessment of ultrastructural characteristics of sciatic nerve from diabetic mice treated with MSC, however, showed markedly fewer morphologic alterations relative untreated diabetic mice. The sciatic nerve of MSC-treated mice presented myelin fibers of various sizes and with proportional caliber sheaths surrounding the axon, myelin sheath with regular contours, and the presence of numerous unmyelinated fibers with homogeneous distribution (Fig. 2e, f). In addition, diabetic mice presented an increased percentage of myelin fibers with morphological alterations, in particular myelin infoldings (Fig. 2g, h). These morphological abnormalities, however, were completely reversed by MSC treatment (*p* < 0.05).

### MSC transplantation reduces morphometric alterations of myelinated and unmyelinated fibers of the sciatic nerve from diabetic mice

**Fig. 3:**
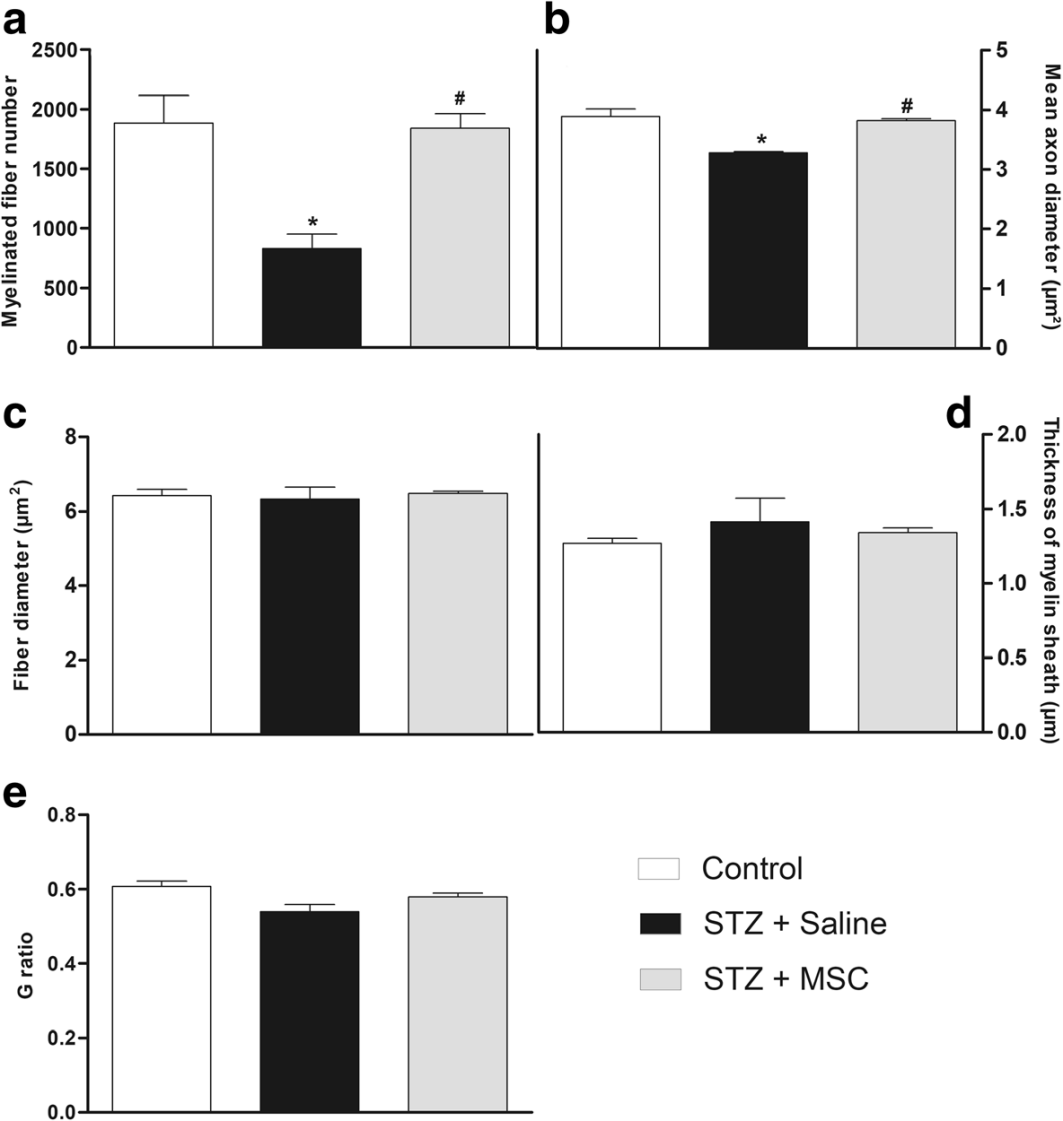
Effects of MSC on the morphometry of sciatic nerve myelinic fibers from mice with diabetic neuropathy. Morphometric analyses of sciatic nerve from non-diabetic mice (control group), diabetic mice treated with saline (STZ + saline), and diabetic mice treated with MSC (1 × 10^6^; STZ + MSC), performed 12 weeks after neuropathy induction. Graphs show **a** myelinated fibers number, **b** mean axon diameter, **c** fiber diameter, **d** thickness of myelin sheath, and **e** G-ratio (ratio axon/nerve fiber diameter). Data are expressed as means ± SEM; *n* = 3 mice per group. *Statistical significance compared to control group (*p* < 0.05). ^#^Statistical significance compared to STZ + saline group (*p* < 0.05). One-way ANOVA followed by Tukey post-test

Different morphometric parameters obtained from the analysis of mouse sciatic nerve myelin fibers were evaluated (Fig. 3). Panels c–e show that the fiber diameter mean, myelin sheath thickness, and *G* ratio (ratio axon/nerve fiber diameter) were not different between diabetic and non-diabetic mice. On the other hand, the number of myelin fibers (panel a) and axon diameter (panel b) was decreased in diabetic neuropathy mice compared to non-diabetic mice (*p* < 0.05). Importantly, MSC transplantation was able to prevent these morphological alterations suggestive of axonal atrophy, which is associated with the diabetic neuropathy evolution.

**Fig. 4:**
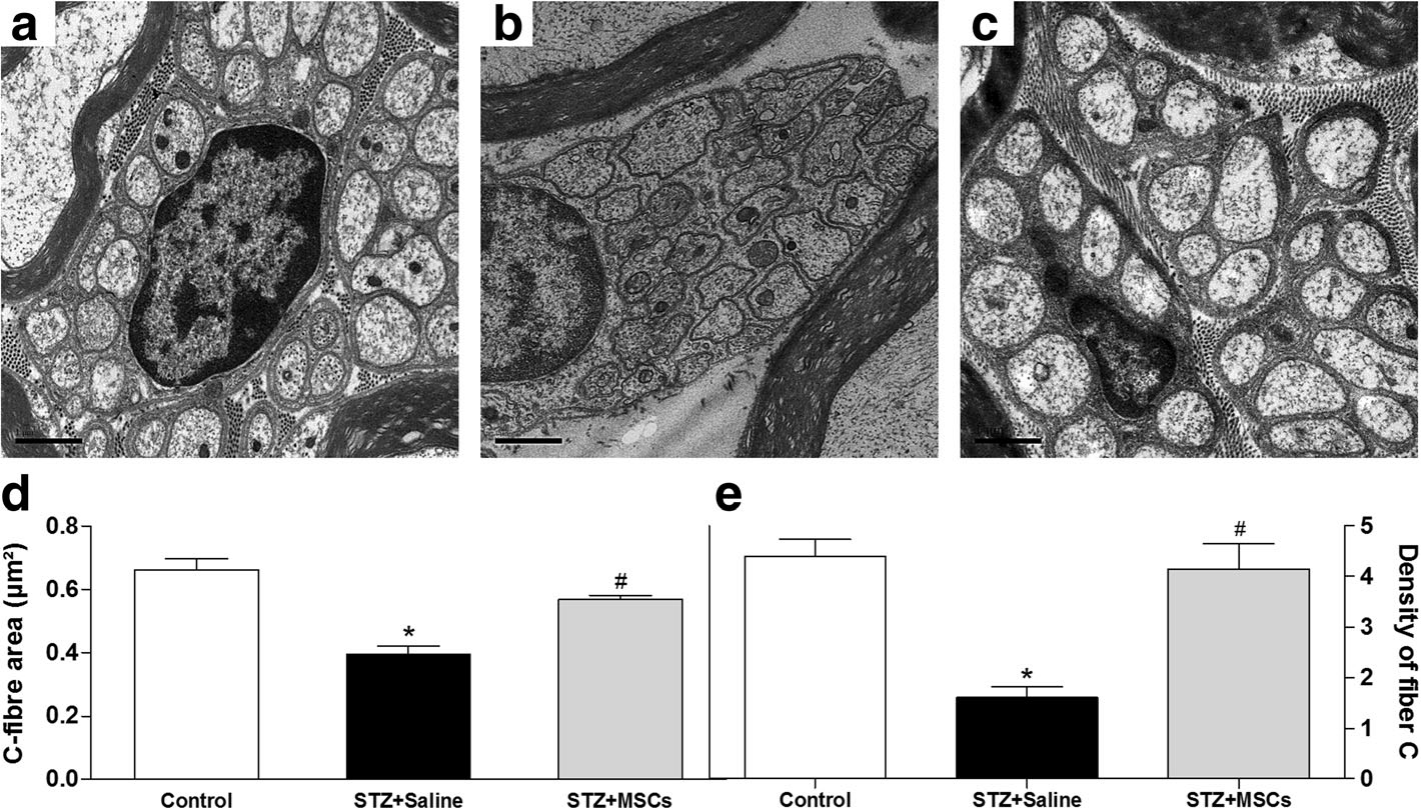
Effects of MSC on morphology and morphometry of C fibers of the sciatic nerve from mice with diabetic neuropathy. Electron microscopy of sciatic nerve cross-sections from non-diabetic mice (panel **a**, control), diabetic mice treated with saline (panel **b**, STZ + saline), and diabetic mice treated with MSC (1 × 10^6^ panel **c**; STZ + MSC), performed 12 weeks after neuropathy induction. Scale bar = 0.5 μm. Ultrastructural analysis of the sciatic nerve showed the effects of MSC treatment on the area (panel **d** and density (panel **e**) of the fiber C of the sciatic nerve of mice with diabetic peripheral neuropathy. Data are expressed as means ± SEM; *n* = 3 mice per group. *Statistical significance compared to control group (*p* < 0.05). ^#^Statistical significance compared to STZ + saline group (*p* < 0.05). One-way ANOVA followed by Tukey post-test

Figure 4 shows the morphology and morphometry of unmyelinated C fiber mouse sciatic nerves. Panels a–c are the representative electromicrographs of nucleated Remak bundle of non-diabetic, diabetic, and MSC-treated diabetic mice. Diabetic mice presented shrunken C fibers, lower C fiber area, and lower C fiber density than non-diabetic control mice (Fig. 4b–e, *p* < 0.01). Morphological and morphometric parameters of C fibers in diabetic mice treated with MSC did not differ from those observed in non-diabetic mice.

### Effects of MSC transplantation on glial cell expression in the dorsal horn of the spinal cord of neuropathic mice

**Fig. 5:**
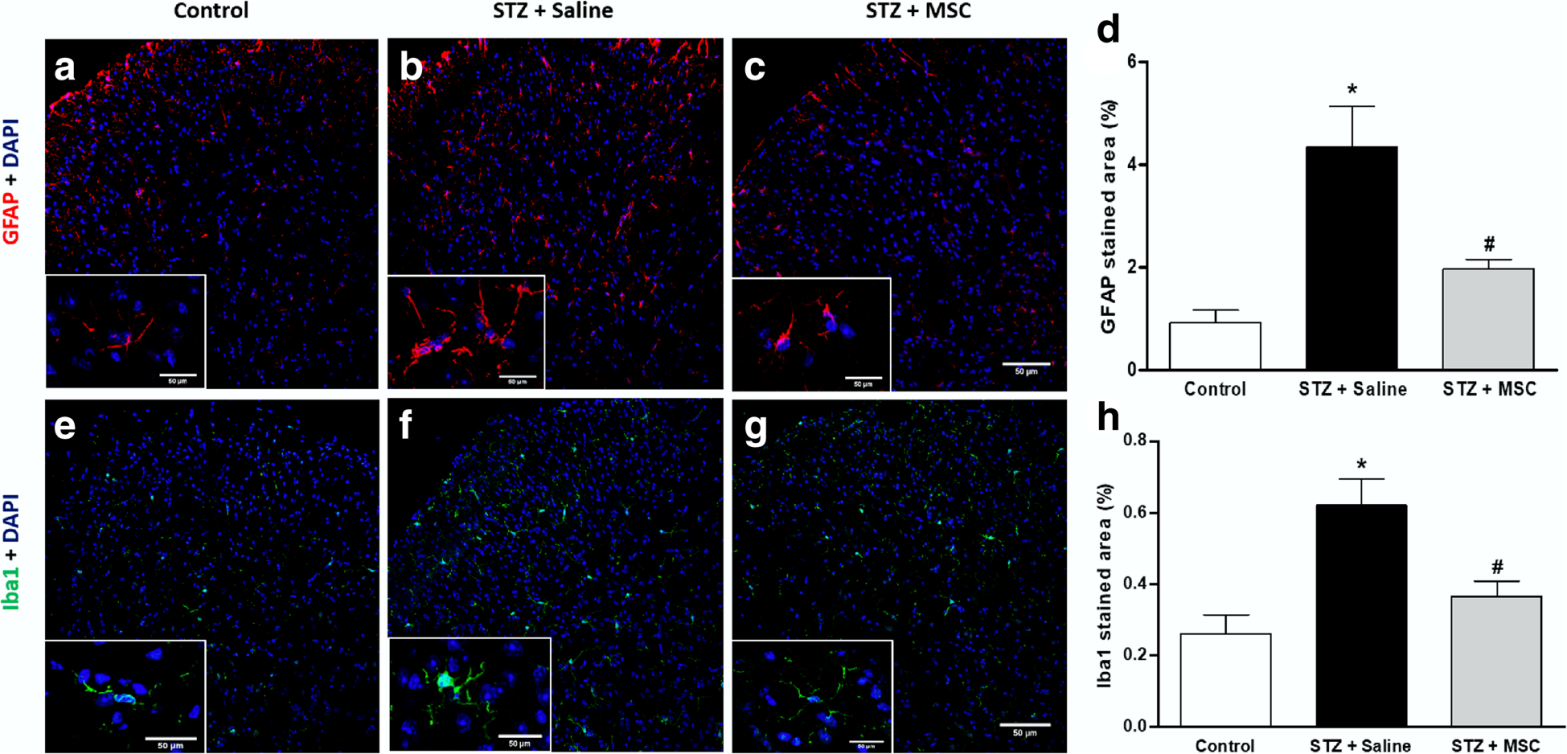
MSC transplantation reduce glial cell expression in the dorsal horn of the spinal cord of neuropathic mice. Eight weeks after the treatment with MSC (1 × 10^6^; STZ + MSC) or saline (STZ + saline), glial cell expression in the spinal cord of neuropathic mice was evaluated. Control non-diabetic group received saline instead of streptozotocin. Representative photomicrographs of histological sections of the mouse spinal cord immunolabeled with GFAP (**a**–**c**) or Iba1 (**e**–**g**). Images are at 200×. Insets demonstrate representative photomicrographs of GFAP or Iba1 positive cells under magnification of 400×. Scale bar = 50 μm. Panels **d** and **h** show the quantitative analysis of the percentage area GFAP and Iba1 positive in the spinal dorsal horn, respectively. Data are expressed as means ± SEM; *n* = 3 mice per group. *Statistical significance compared to the remaining groups (*p* < 0.05). One-way ANOVA followed by Tukey post-test

Activation of glial cells in the spinal cord is a key event in the development and maintenance of sensory neuropathy. Considering that a single MSC transplantation resulted in the complete reversion of sensorial dysfunction in diabetic mice, a possible modulatory action of MSC on glial cell expression during neuropathy was next evaluated. To assess microglia and astrocyte expression in the spinal cord, immunostaining for Iba1 and GFAP was performed, and representative photomicrographs of histological sections of the spinal cord of mice are shown in Fig. 5. Saline-treated diabetic mice showed higher immunoreactivity for Iba1 and GFAP compared to the non-diabetic control group (*p* < 0.05). Treatment of diabetic mice with MSC significantly reduced the spinal immunoreactivity for Iba1 and GFAP, when compared to untreated diabetic mice.

### MSC treatment reduces oxidative/nitrosative stress biomarkers in the spinal cord of diabetic mice

**Fig. 6:**
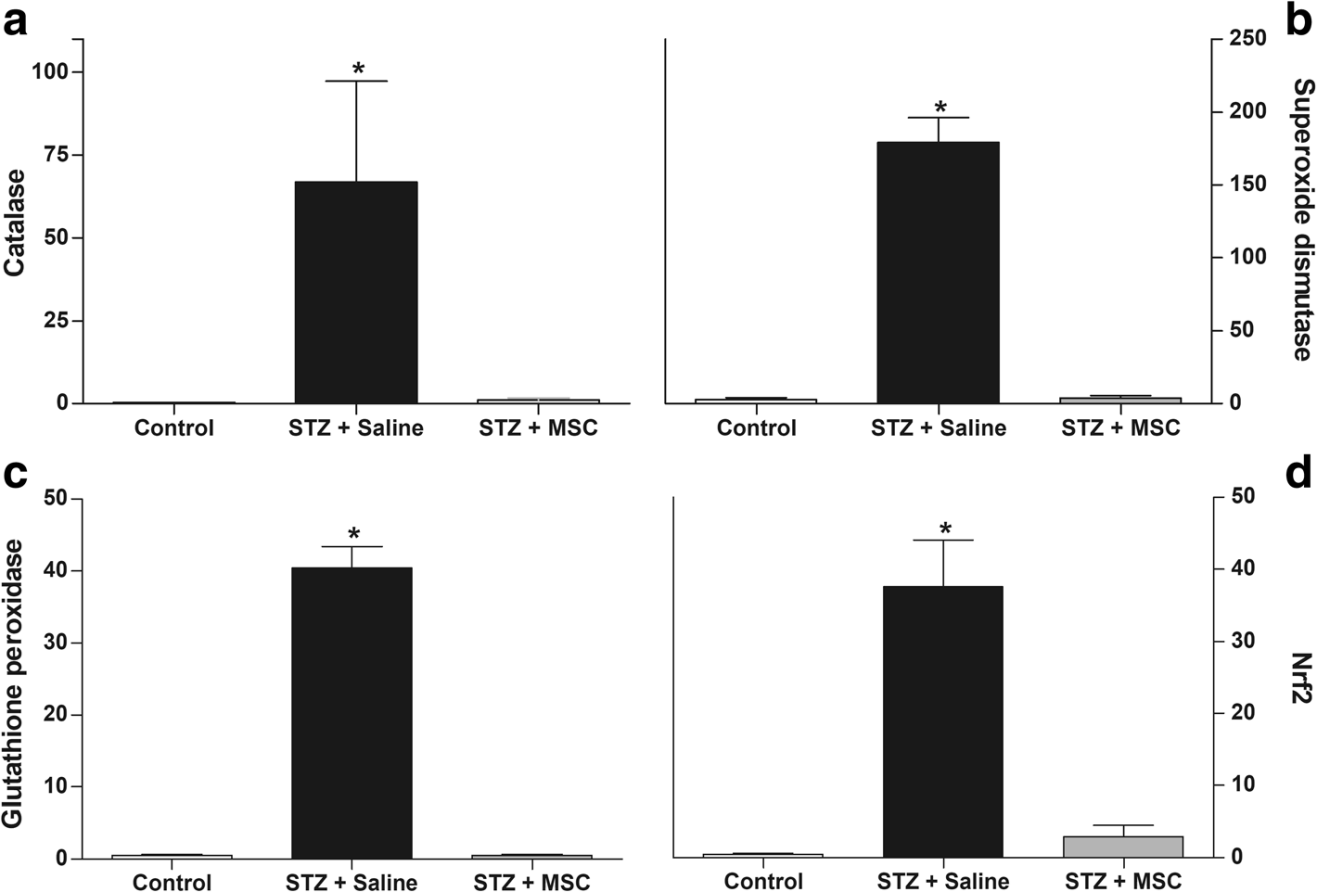
Effect of MSC on the antioxidant profile in the spinal cord of mice with diabetic neuropathy. Four weeks after the neuropathy induction, mice were treated with MSC (1 × 10^6^; STZ + MSC) or saline (STZ + saline) by endovenous route. Control non-diabetic group received saline instead of streptozotocin. The spinal levels of mRNA were measured by RT-qPCR 8 weeks after treatment. Panels show the spinal levels of catalase mRNA (**a**), superoxide dismutase mRNA (**b**), glutathione peroxidase mRNA (**c**), and Nrf2 mRNA (**d**). Data are expressed as means ± SEM; *n* = 6 mice per group. *Statistical significance compared to the remaining groups (*p* < 0.001). One-way ANOVA followed by Tukey’s multiple comparison test

RT-qPCR analysis for selected key molecules showed an antioxidant profile in the spinal cord of mice with diabetic neuropathy. Diabetic mice presented higher values of catalase (Fig. 6a), superoxide dismutase (Fig. 6b), glutathione peroxidase (Fig. 6c), and Nrf2 (Fig. 6d) mRNA in the spinal cord compared to non-diabetic mice (*p* < 0.01). Eight weeks after transplantation, diabetic mice treated with MSC showed reduced mRNA expression of these antioxidant factors in the spinal cord, compared to saline-treated diabetic mice.

**Fig. 7:**
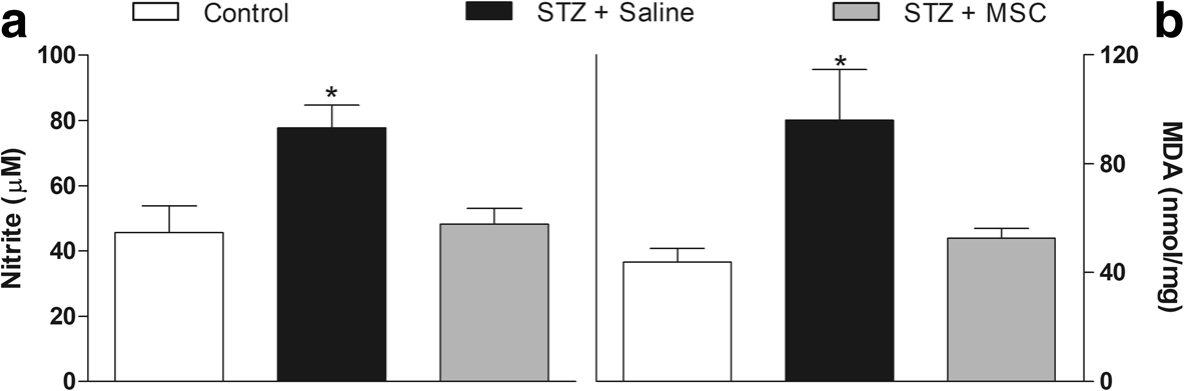
MSC reduces nitrite and MDA levels in the spinal cord of mice with diabetic neuropathy. Four weeks after neuropathy induction, mice were treated with MSC (1 × 10^6^; STZ + MSC) or saline (STZ + saline) by endovenous route. Control non-diabetic group received saline instead of streptozotocin. The spinal levels of nitrite (**a**) and MDA (**b**) were measured 8 weeks after treatments. Data are expressed as means ± SEM; *n* = 6 mice per group. *Statistical significance compared to the remaining groups (*p* < 0.05). One-way ANOVA followed by Tukey’s multiple comparison test

Next, the effects of MSC on nitrosative stress and lipid peroxidation were investigated by measuring the tissue levels of nitrite and MDA in the spinal cord 8 weeks after transplantation. Nitrite (Fig. 7a) and MDA (Fig. 7b) levels, significantly elevated in the spinal cord of diabetic mice when compared to the control non-diabetic group (*p* < 0.05), were significantly reduced in MSC treated in the spinal cord of diabetic mice.

### A single transplantation of MSC modulates the pattern of spinal cytokine production in diabetic mice

**Fig. 8:**
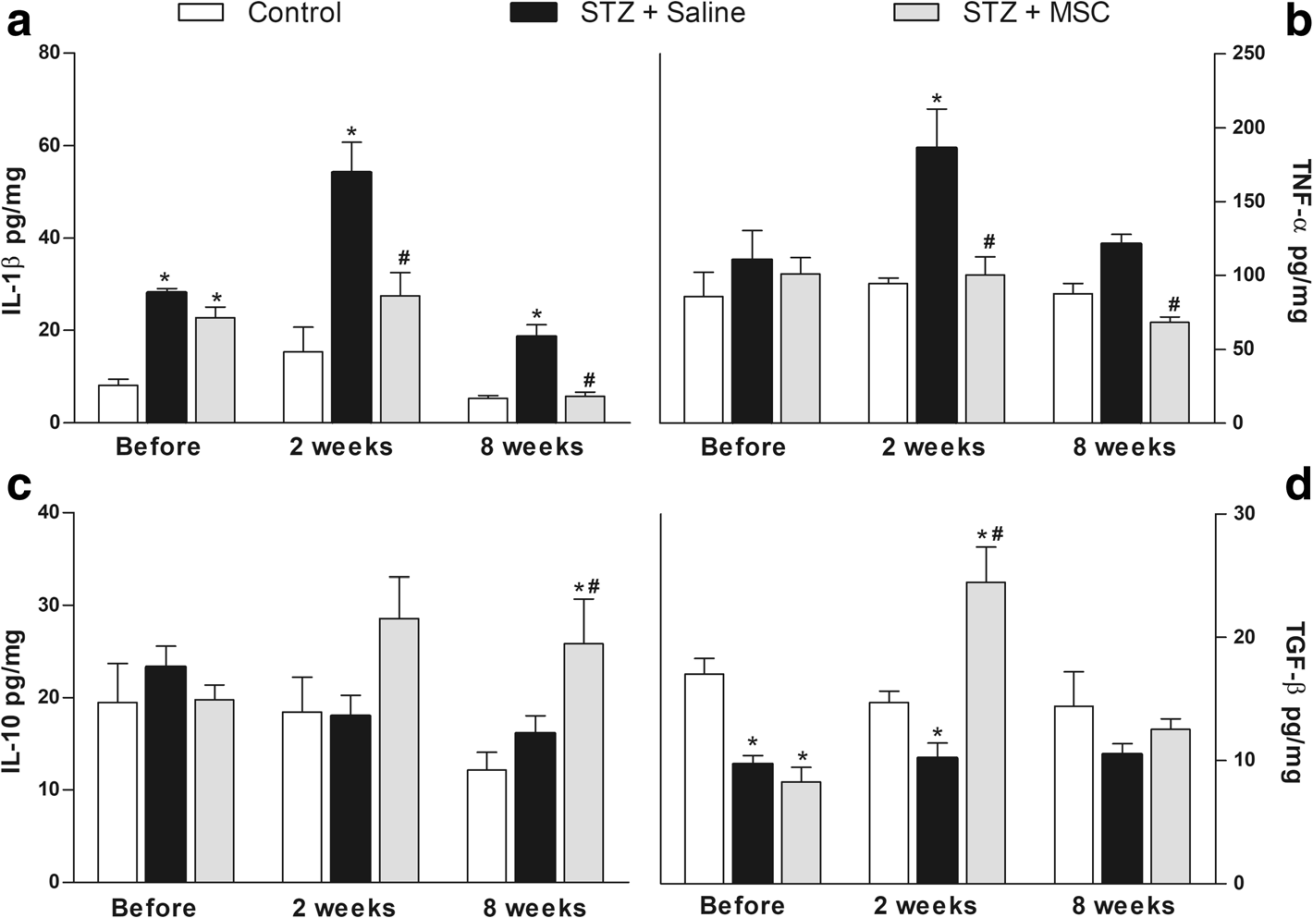
MSC transplantation modulates cytokine expression in the spinal cord of mice with diabetic neuropathy. Four weeks after the neuropathy induction, mice were treated with MSC (1 × 10^6^; STZ + MSC) or saline (STZ + saline) via endovenous route. Control group received saline instead of streptozotocin. Spinal cytokine levels were evaluated before, 2 and 8 weeks after treatments. Panels show the spinal levels of **a** interleukin-1β (IL-1β), **b** tumor necrosis factor-α (TNF-α), **c** interleukin-10 (IL-10), and **d** transforming growth factor-β (TGF-β). The results are expressed as picograms of cytokine per milligram of protein. Data are expressed as means ± SEM; *n* = 6 mice per group. *Statistically significant as compared to the control group (*p* < 0.001). ^#^Statistical significance in relation to the STZ + saline group (*p* < 0.05). One-way ANOVA followed by Tukey’s multiple comparison test

Next, a possible modulatory action of MSC on spinal cytokine production during diabetic neuropathy was evaluated. Levels of IL-1β, TNF-α, IL-10, and TGF-β in the spinal cord (L5-L4) were evaluated before, 2 and 8 weeks after treatments (Fig. 8). ELISA analysis demonstrated that saline-treated diabetic mice exhibited upregulation of IL-1β (Fig. 8a) and TNF-α (Fig. 8b) in the spinal cord, relative to non-diabetic control mice (*p* < 0.001). Diabetic mice also presented reduction of the spinal cord TGF-β (Fig. 8d), but not IL-10 (Fig. 8c), levels. The levels of IL-1β and TNF-α were reduced, while IL-10 and TGF-β were enhanced, in the spinal cord of neuropathic mice treated with MSC (*p* < 0.05).

### MSC transplantation reduces galactin-3 expression in the dorsal horn of the spinal cord of neuropathic mice

**Fig. 9:**
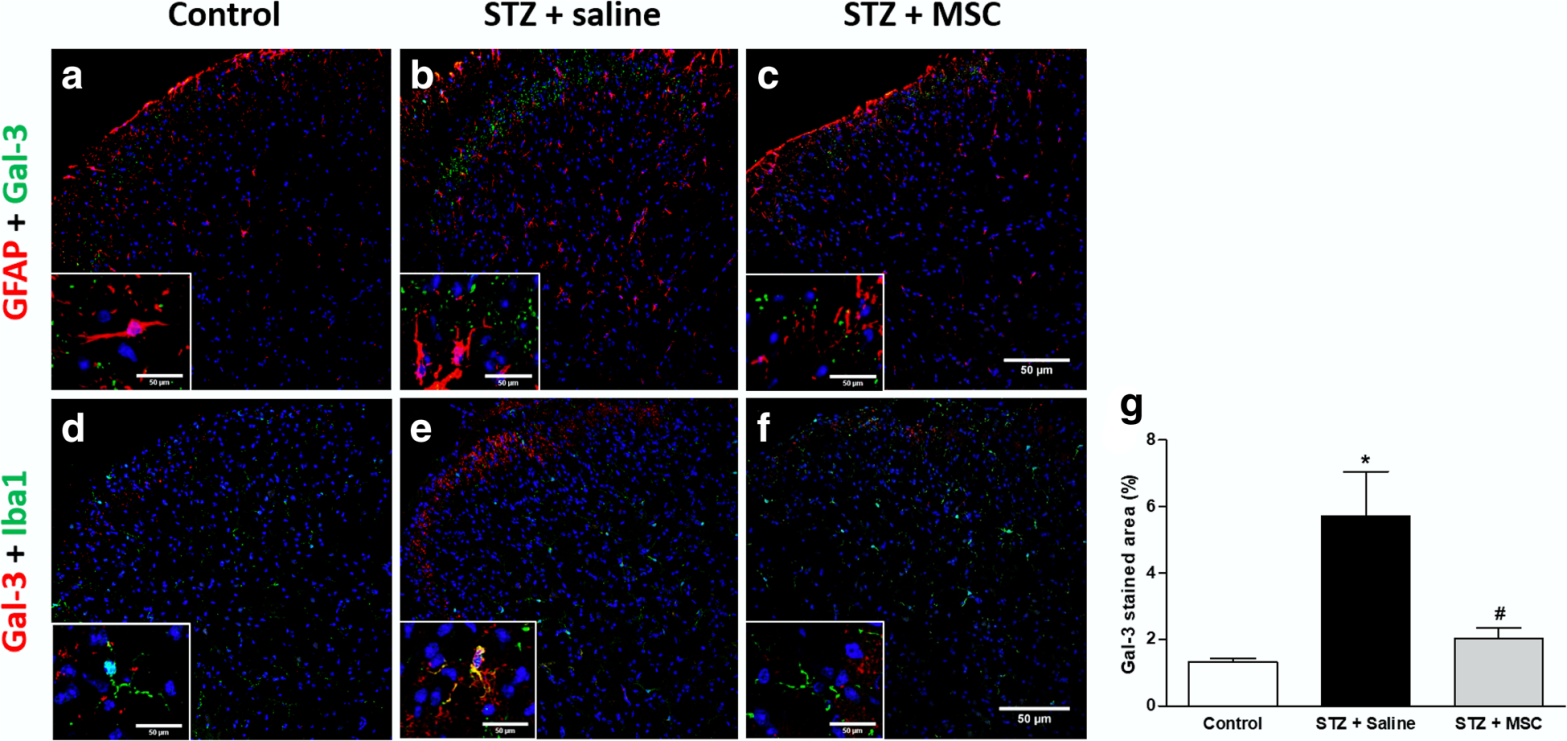
MSC transplantation reduces galectin-3 expression in the dorsal horn of the spinal cord of neuropathic mice. Eight weeks after the treatment with MSC (1 × 10^6^; STZ + MSC) or saline (STZ + saline), the galectin-3 (Gal-3) expression in the spinal cord of neuropathic mice was evaluated. Control non-diabetic group received saline instead of streptozotocin. Representative photomicrographs of spinal cord sections co-stained with Gal-3 and GFAP (**a**–**c**) or Gal-3 and Iba-1 (**d**–**f**) (200×). Insets (400×) evidenced the presence of co-staining for Gal-3 and Iba1 (**e**) in saline-treated diabetic mice and the absence of Gal-3 and GFAP co-stained cells (**a**–**c**). Scale bar = 50 μm. Panel **g** shows the quantitative analysis of the percentage area Gal-3 positive in the spinal dorsal horn. Data are expressed as means ± SEM; *n* = 3 mice per group. *Statistically significant as compared to the control group (*p* < 0.05). ^#^Statistically significant as compared to the STZ + saline group (*p* < 0.05). One-way ANOVA followed by Tukey’s multiple comparison test

The expression profile of galectin-3 in the spinal cord of diabetic mice was investigated by immunofluorescence, 8 weeks after the treatment with MSC (1 × 10^6^; STZ + MSC) or saline (STZ+ saline). Control non-diabetic group received saline instead of streptozotocin. Figure 9 shows representative images of the co-labeling for galectin-3 and Iba-1 (A-F), and galectin-3 and GFAP (G-I). Spinal cord sections of saline-treated diabetic mice showed higher immunoreactivity for galectin-3 in the dorsal horn, compared to the non-diabetic control group (*p* < 0.05; panel j). The treatment of diabetic mice with MSC completely reversed the increased spinal immunoreactivity for galectin-3. The co-labeling studies demonstrated the presence of some galectin-3^+^ microglial cells (Iba-1 positive cells) in saline-treated diabetic mice. Cells positive for galectin-3 and GFAP, a marker of activated astrocyte, were not observed.

### Transplanted MSC tracking

**Fig. 10:**
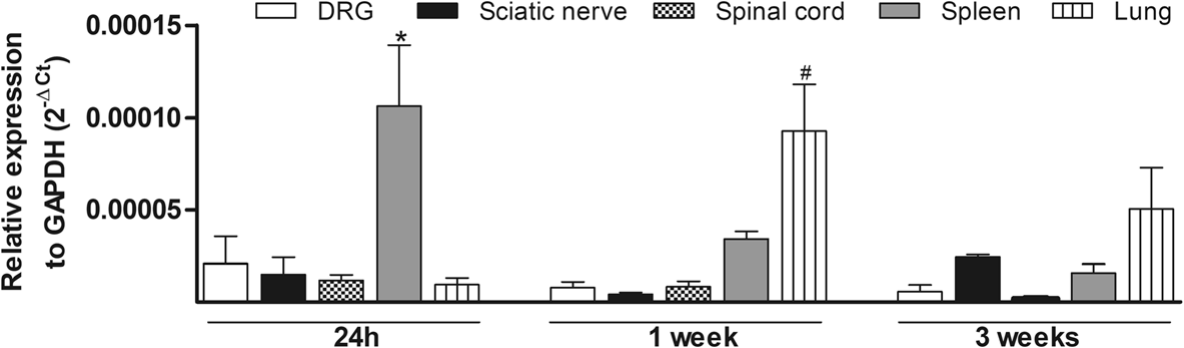
Transplanted MSC tracking. The levels of GFP mRNA were measured by RT-qPCR 24 h, 1 and 3 weeks after MSC treatment. MSC were obtained from GFP transgenic C57Bl/6 mice. Bars show GFP mRNA expression on the dorsal root ganglion (DRG), sciatic nerve, spinal cord, spleen, and lung of transplanted neuropathic mice. Data are expressed as means ± SEM; *n* = 5 per group for each timepoint. *Statistical significance compared to the remaining groups at the same timepoint (*p* < 0.005). ^#^Statistical significance compared to the DRG, sciatic nerve, and spinal cord groups at the same timepoint (*p* < 0.005). One-way ANOVA followed by Tukey’s multiple comparison test

RT-qPCR analysis for GFP identified the location of transplanted MSC in neuropathic mice 24 h, 1 and 3 weeks after transplantation. Samples of the lung, spleen, sciatic nerve, dorsal root ganglion, and spinal cord were analyzed. Although GFP expression was detected at low levels in all tissues evaluated, it was significantly higher in the spleen and lung samples (Fig. 10). Twenty-four hours after MSC treatment, GFP mRNA levels were increased in spleen samples (*p* < 0.05), whereas in the 1-week timepoint showed an increased in the lung sample (*p* < 0.05). Three weeks after transplantation, the level of GFP expression was less in the tissues evaluated.

### Conditioned medium from MSC reduces the sensorial dysfunction in diabetic neuropathic mice

**Fig. 11:**
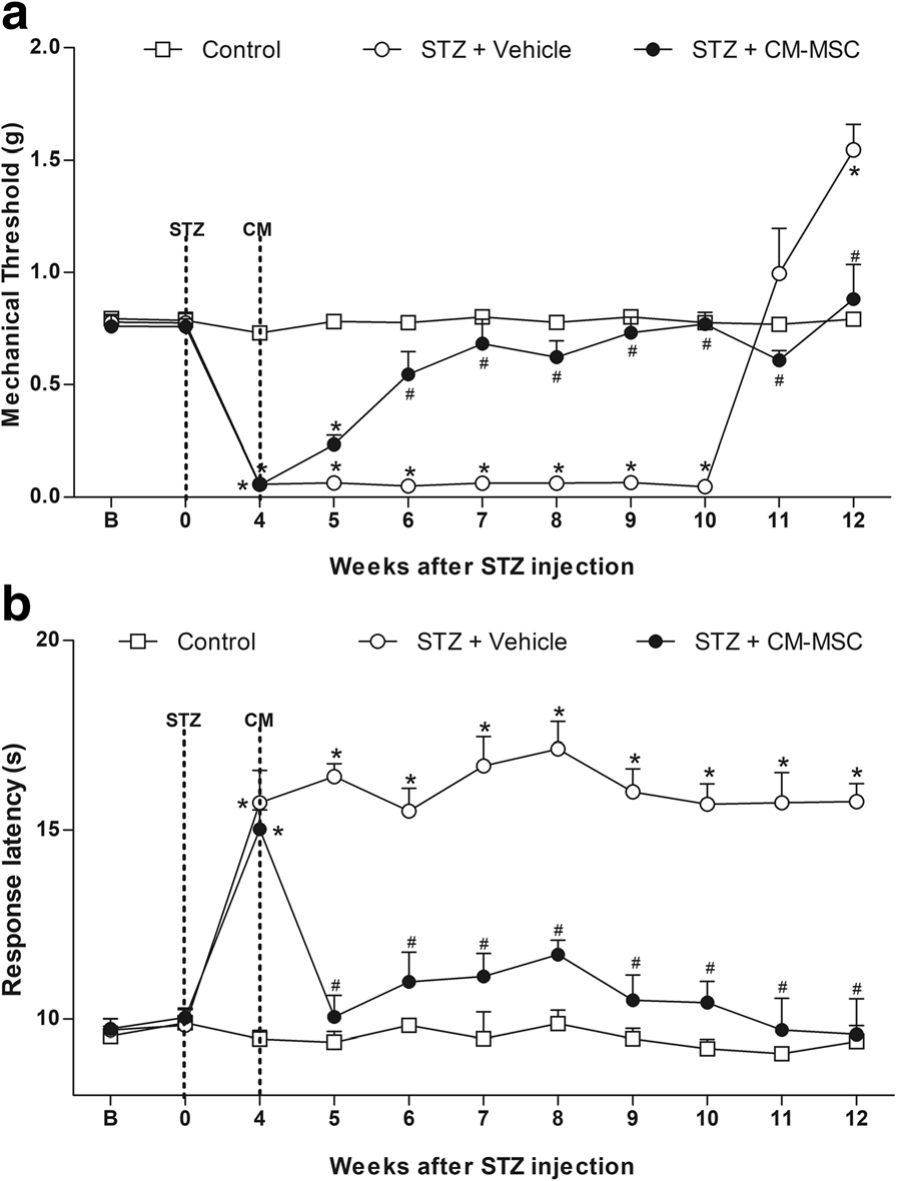
Effect of CM-MSC on pain-like behaviors of mice with diabetic neuropathy. **a** Mechanical nociceptive thresholds: ordinates represent the filament weight (g) in which the animal responds in 50% of presentations. **b** Thermal nociceptive threshold: the axis of ordinates represents the time (seconds) the animal takes to withdraw its paw. The nociceptive thresholds were assessed in the paw of each mouse before (**b**) and after the model induction with streptozotocin (STZ; week 0). Control group represents mice without diabetic neuropathy, in which saline was administered instead of streptozotocin. Four weeks after induction, mice were treated via endovenous route with conditioned medium from MSC cultures (STZ + CM-MSC; 100 μL) or vehicle (STZ + vehicle; 100 μL). Data are expressed as means ± SEM; *n* = 6 mice per group. *Statistical significance relative to the control group (*p* < 0.001). ^#^Statistical significance relative to the STZ + vehicle group (*p* < 0.001), as determined by two-way ANOVA followed by Bonferroni post-test

In order to corroborate the hypothesis that the MSC-induced beneficial effects on neuropathic pain correlate with paracrine actions, the effects of CM-MSC on diabetic sensory neuropathy were evaluated. Neuropathic mice were treated with CM-MSC or vehicle 4 weeks after the induction of diabetes, when the sensorial neuropathy was fully established. Two weeks after administration, CM-MSC-treated neuropathic mice showed complete reversion of mechanical allodynia, and this antinociceptive effect was maintained throughout the experimental period (Fig. 11a; *p* < 0.001). CM-MSC treatment reverted the heat hypoalgesia of neuropathic mice from 1 week after administration until the completion of the evaluation period (Fig. 11b; *p* < 0.001).

## Discussion

Diabetic neuropathy is associated with sensory symptoms, such as pain and enhanced/diminished sensation, for which there is no effective therapy, ultimately leading to considerably reducing the patient’s quality of life. The present study demonstrated that a single MSC administration was able to reverse the sensory diabetic neuropathy through modulation of crucial events during neuropathy maintenance, in both the peripheral and central nervous systems, highlighting the cell therapy potential for the neuropathy treatment. In addition to the previously described effects of MSC on peripheral nerves, the present study described the modulatory effect of MSC in the local microenvironment of the diabetic mouse spinal cord.

The development of sensory neuropathy was confirmed by using established behavioral assays, which detected the characteristic mechanical allodynia and heat hypoalgesia observed in diabetic neuropathy in mice [19]. About 40% of individuals with DN manifest mechanical allodynia [39], progressing to a loss of sensory perception in the late disease stages, which is closely related to cases of limb ulceration and amputation [40]. In the present study, mechanical allodynia was completely present in mice, 4 weeks after STZ administration, and progressed to loss of tactile sensitivity 12 weeks after STZ, indicating a strong equivalence between the model and the clinical manifestation. The MSC transplantation completely reverted the signs of sensory-induced neuropathy in the STZ-diabetic mouse model. In fact, behavior sensory neuropathy improvements in diabetic mice with MSC treatments have previously been demonstrated [9, 19, 14]. Recent evidence indicate that MSC may act as biologic “pumps” by releasing analgesic molecules responsible to mediate the therapeutic effects of these cells in neuropathic pain [41]. On the other hand, in the present study, the reversal of sensory neuropathy following a single injection of MSC was maintained throughout the evaluation period, an effect that is not attained by analgesic substances. This effect profile suggests that a reparative action, more than just an analgesic effect, may result after MSC treatment. This view is supported by literature data showing that MSC treatment induces angiogenesis, neurotrophic effects, and restoration of myelination in peripheral nerves of diabetic animals [9, 11–13]. Further ultrastructural analyses of myelinated and unmyelinated fibers of the sciatic nerve, presented here, support this hypothesis. Morphological and morphometric analysis of the sciatic nerve showed significant differences between sensory fibers of diabetic and non-diabetic mice, such as the presence of degenerative changes in axons, morphological alterations in the myelin sheath, and lower unmyelinated C fiber area and density. In MSC-treated diabetic mice, all these morphologic alterations, considered to be hallmarks of DN, were markedly reduced, indicating that the cell therapy restored sensory fiber structures in diabetic nerves. The present data are consistent with the recent study of Han and co-workers, showing that MSC restores the ultrastructure of myelinated fibers in diabetic nerve, while giving the first evidence of the restorative action of MSC on unmyelinated C fibers [13]. Morphological and functional alterations of both Aδ and C nociceptive fibers present in peripheral nerves have been proposed to explain the changes in sensitivity and pain during diabetic neuropathy. Maintaining hyperglycemia for prolonged periods leads to sensory nerve damage, thus modifying the underlying neural transmission pattern, resulting in the loss or gain of sensitivity to painful stimuli [42, 43]. A relationship between reduced number of sensory fibers in peripheral nerves and thermal/mechanical hyposensitivity in diabetic mice was proposed by Lennertz and colleagues [44] and reinforced here. Therefore, it is possible that the long-lasting effect of MSC reversing behavioral sensory neuropathy stems from the reestablishment of ultrastructures within peripheral diabetic nerves. On the other hand, the mechanisms by which MSC reduce morphological alterations of nociceptive fibers of the peripheral nerves during neuropathy are not fully comprehended. It has been proposed that MSC promote the repair of peripheral nerves in diabetic animals through paracrine actions of neurotrophic and angiogenic factors, such as vascular endothelial growth factor (VEGF), nerve growth factor (NGF), and hepatocyte growth factor (HGF), secreted by these cells [9, 11–13]. However, the contribution of these growth factors to MSC-induced reparative action on peripheral nerve during diabetic neuropathy still needs to be further investigated.

The mechanisms underlying DN maintenance involve multiple biochemical and cellular alterations in the central nervous system, which may be a contributory factor in the refractory nature of the diabetic neuropathy. Hyperglycemia disturbs spinal cord homeostasis, while high glucose concentrations are responsible for inducing metabolic and enzymatic abnormalities, and disrupts the functions of cell mitochondria inducing the overproduction of reactive oxygen/nitrogen species (ROS/RNS) and oxidative stress [22, 45–48]. In addition, hyperglycemia and the subsequent formation of ROS/RNS induce the activation of spinal microglia [22], triggering spinal neuroinflammatory cascades that have been considered a pivotal event of sensory neuropathy [23, 47, 49]. In line with this idea, the present study demonstrated that GFAP and Iba-1, markers of activated astrocyte and activated microglia, respectively, were highly expressed in the dorsal horn of the spinal cord in diabetic mice. Importantly, MSC transplantation significantly reduced the staining for GFAP and Iba-1, indicating a suppression of the spinal activation of astrocytes and microglia by MSC. Although the inhibitory effect of mesenchymal cells on spinal glial cell activation has been previously demonstrated in experimental neuropathic pain associated with spinal cord injury [50] and spinal nerve ligation [49], the results from our study are the first demonstration of this phenomenon in diabetic neuropathy.

Considering the contribution of oxidative stress to the glial cell activation, the redox homeostasis in the spinal cord was also investigated. Nitrosative stress and lipid peroxidation, indicated by high levels of nitrite and MDA in the spinal cord, were evidenced in diabetic mice. The high production of ROS/RNS is controlled by the activation of cell antioxidant defense system, which includes a range of antioxidant enzymes [51]. In fact, qRT-PCR data showed upregulation of catalase, superoxide dismutase, glutathione peroxidase, and Nrf2 mRNA, indicating the presence of oxidative stress in the spinal cord microenvironment of diabetic mice. Importantly, MSC treatment reduced both the ROS/RNS levels and the activation of the antioxidant defense system in the spinal cord of diabetic mice, suggesting that a single MSC administration is able to promote the reestablishment of redox homeostasis in the spinal cord. The antioxidant properties of stem cells have been previously described by in vitro [52–54] and in vivo [55, 56] studies. In line with the present data, MSC were found to influence the spinal redox context in models of traumatic spinal cord injury [56] and amyotrophic lateral sclerosis [57].

There is emerging evidence for the causal role of oxidative stress underlying activation of glial cells in diabetic neuropathy [58]. During neuropathic states, glial cells in the spinal cord are activated, accompanied by a wide cascade release of neuroactive molecules and pro-inflammatory cytokines, such as IL-1β and TNF-α, which have been directly implicated in the excitability pattern change of spinal neurons and sensory neuropathy [22, 47, 59]. In line with this concept, in the present study, diabetic mice presented enhanced levels of IL-1β and TNF-α in the spinal cord, while MSC treatment inhibited this upregulation, in parallel with its effects on behavioral sensory neuropathy. The modulatory action of MSC on cytokine expression in peripheral tissues of diabetic rodents has been previously demonstrated [12, 19]. In addition, MSC administration enhanced the levels of the anti-inflammatory cytokines IL-10 and TGF-β in the spinal cord of neuropathic mice. IL-10 and TGF-β induce therapeutic effects in neuropathic conditions [8, 60, 61], and their role to the antinociception induced by stem cells in neuropathic pain has been proposed [49, 62].

Galectin-3 is a member of the lectin family that binds β-galactosides, which plays a major role in mechanisms of inflammation [63–66]. Since galectin-3 is involved in oxidative stress [67–69] and neuroinflammation in the spinal cord [70, 71], the spinal expression of this lectin was evaluated in diabetic mice. Immunofluorescence analyses showed that galectin-3 was overexpressed in the superficial dorsal horn of the spinal cord of diabetic mice, and this phenomenon was reversed by MSC transplantation. These data highlight the broad inhibitory effect induced by mesenchymal cells in the spinal neuroinflammatory response, triggered by hyperglycemia/diabetes.

It has been proposed that the therapeutic effects of MSC during neuropathic conditions are related to the paracrine action of these cells [7, 9, 26]. Data from the present work corroborate this hypothesis. By detecting levels of GFP mRNA in different tissues of neuropathic mice, it was possible to demonstrate that some transplanted MSC were localized in the lung and spleen, with minimal retention in the sciatic nerve, dorsal root ganglion, and spinal cord. This result indicates that the MSC-induced beneficial effects during diabetic neuropathy are independent of the presence of significant amount of the transplanted cells at the lesion site. In addition, a single systemic administration of the conditioned medium from MSC culture induced long-lasting antinociceptive effect in neuropathic mice. Interestingly, the profile and magnitude of the CM-MSC-induced beneficial effects on sensory neuropathy were similar to those induced by MSC transplantation. In fact, the remarkable therapeutic potential of CM-MSC has been consistently demonstrated in several experimental models, including in nervous system disorders and painful conditions [26, 72–75]. These results corroborate the hypothesis that paracrine action of MSC is important to the therapeutic effects of these cells during diabetic neuropathy. Nevertheless, the complex mechanisms by which MSC reduce sensory neuropathy, probably involving a broad spectrum of soluble factors and extracellular vesicles secreted by MSC, need to be further investigated.

## Conclusions

Taken together, our data indicate that, in addition to the beneficial actions of MSC in the peripheral nervous system, blocking the spinal neuroinflammatory cascade is a possible mechanism by which mesenchymal stem cells reduce the signs of sensorial diabetic neuropathy.

## Abbreviations

DMEM: Dulbecco’s modified Eagle’s medium
DN: Diabetic neuropathy
gal-3: Galectin-3
i.p.: Intraperitoneal
IL-10: Interleukin-10
IL-1β: Interleukin-1β
MDA: Malondialdehyde
MSC: Mesenchymal stem/stromal cells
Nrf2: Nuclear factor erythroid 2-related factor 2
PBS: Phosphate-buffered saline
qRT-PCR: Real-time quantitative polymerase chain reaction
ROS/RNS: Reactive oxygen/nitrogen species
STZ: Streptozotocin
TGF-β: Transforming growth factor-β
TNF-α: Tumor necrosis factor-α
